# Timing of State and Territorial COVID-19 Stay-at-Home Orders and Changes in Population Movement — United States, March 1–May 31, 2020

**DOI:** 10.15585/mmwr.mm6935a2

**Published:** 2020-09-04

**Authors:** Amanda Moreland, Christine Herlihy, Michael A. Tynan, Gregory Sunshine, Russell F. McCord, Charity Hilton, Jason Poovey, Angela K. Werner, Christopher D. Jones, Erika B. Fulmer, Adi V. Gundlapalli, Heather Strosnider, Aaron Potvien, Macarena C. García, Sally Honeycutt, Grant Baldwin, CDC Public Health Law Program, CDC COVID-19 Response Team, Mitigation Policy Analysis Unit, CDC Public Health Law Program, Catherine Clodfelter, Mara Howard-Williams, Gi Jeong, Lisa Landsman, Julia Shelburne, CDC COVID-19 Response Team, Mitigation Policy Analysis Unit, Amanda Brown, Ryan Cramer, Siobhan Gilchrist, Rachel Hulkower, Alexa Limeres, Adebola Popoola

**Affiliations:** ^1^CDC Public Health Law Program; ^2^Georgia Tech Research Institute, Atlanta, Georgia; ^3^CDC COVID-19 Response Team.; CDC Public Health Law Program; CDC Public Health Law Program; CDC Public Health Law Program; CDC Public Health Law Program; CDC Public Health Law Program.; CDC COVID-19 Response Team, Mitigation Policy Analysis Unit; CDC COVID-19 Response Team, Mitigation Policy Analysis Unit; CDC COVID-19 Response Team, Mitigation Policy Analysis Unit; CDC COVID-19 Response Team, Mitigation Policy Analysis Unit; CDC COVID-19 Response Team, Mitigation Policy Analysis Unit; CDC COVID-19 Response Team, Mitigation Policy Analysis Unit.

SARS-CoV-2, the virus that causes coronavirus disease 2019 (COVID-19), is thought to spread from person to person primarily by the respiratory route and mainly through close contact (*1*). Community mitigation strategies can lower the risk for disease transmission by limiting or preventing person-to-person interactions (*2*). U.S. states and territories began implementing various community mitigation policies in March 2020. One widely implemented strategy was the issuance of orders requiring persons to stay home, resulting in decreased population movement in some jurisdictions (*3*). Each state or territory has authority to enact its own laws and policies to protect the public’s health, and jurisdictions varied widely in the type and timing of orders issued related to stay-at-home requirements. To identify the broader impact of these stay-at-home orders, using publicly accessible, anonymized location data from mobile devices, CDC and the Georgia Tech Research Institute analyzed changes in population movement relative to stay-at-home orders issued during March 1–May 31, 2020, by all 50 states, the District of Columbia, and five U.S. territories.* During this period, 42 states and territories issued mandatory stay-at-home orders. When counties subject to mandatory state- and territory-issued stay-at-home orders were stratified along rural-urban categories, movement decreased significantly relative to the preorder baseline in all strata. Mandatory stay-at-home orders can help reduce activities associated with the spread of COVID-19, including population movement and close person-to-person contact outside the household.

Data on state and territorial stay-at-home orders were obtained from government websites containing executive or administrative orders or press releases for each jurisdiction. Each order was analyzed and coded into one of five mutually exclusive categories: 1) mandatory for all persons; 2) mandatory only for persons in certain areas of the jurisdiction; 3) mandatory only for persons at increased risk in the jurisdiction; 4) mandatory only for persons at increased risk in certain areas of the jurisdiction; or 5) advisory or recommendation (i.e., nonmandatory). Jurisdictions that did not issue an order were coded as having no state- or territory-issued order.^†^ These data underwent secondary review and quality assurance checks and were published in a freely available data set (*4*).

Publicly accessible, anonymized location data from mobile devices were obtained to estimate county-level raw data regarding movement (*5*). Population movement was estimated by computing the percentage of individual mobile devices (e.g., mobile phones, tablets, or watches) reporting each day that were completely at home (i.e., had not moved beyond a 150-meter radius of its common nighttime location) within a given county, using a 7-day rolling average to smooth each county’s pre- and postorder time series values. This analysis used four types of order index dates, based only on mandatory orders: 1) the start date of each state or territorial stay-at-home order for each county in that jurisdiction; 2) the relaxation or expiration date of each state or territorial stay-at-home order for each county in that jurisdiction; 3) the effective date of the first state-issued stay-at-home order (i.e., California); and 4) the first date a state-issued stay-at-home order ended (i.e., Alaska).^§^

To assess changes in movement when mandatory state or territorial stay-at-home orders went into effect and ended, counties were first stratified along rural-urban categories to ensure that counties with similar population sizes were grouped together.^¶^ A box plot was constructed for each rural-urban category to examine the distribution of county mean percentages of devices at home during the pre- and postorder periods associated with each index date. Because it was not assumed that movement values follow a normal distribution for all counties and periods, a clustered Wilcoxon signed rank test was then performed for each stratum, with counties as clusters, on the constituent counties’ median pre- and postorder values associated with each index date. A lower-tailed test was used for index dates related to the start of state and territorial orders, and an upper-tailed test was used for index dates related to the end of state and territorial orders** (*6*). Strata-level statistical significance was assessed at the 99% confidence level (α = 0.01). Analyses were performed using Python (version 3.6; Python Software Foundation) and R (version 3.5; The R Foundation). This activity was reviewed by CDC and was conducted consistent with applicable federal law and CDC policy.^††^

During March 1–May 31, 42 states and territories issued mandatory stay-at-home orders, affecting 2,355 (73%) of 3,233 U.S. counties (Figure 1). The first territorial order was issued by Puerto Rico (March 15), and the first state order by California (March 19). Eight jurisdictions issued only an advisory order or recommendation to stay home, and six did not issue any stay-at-home orders. Most jurisdictions issued multiple orders during the observation period, and coding varied among individual orders. The duration and termination of each order varied by jurisdiction. During the observation period, 22 jurisdictions transitioned from a mandatory order to an advisory order, 11 rescinded or allowed orders to expire without extending, and the order in one jurisdiction was ruled invalid by the state’s supreme court.^§§^ The first state to rescind or allow a stay-at-home order to expire was Alaska (April 24). Eight jurisdictions had mandatory orders applicable to at least some part of the population that extended beyond May 31.

**FIGURE 1:**
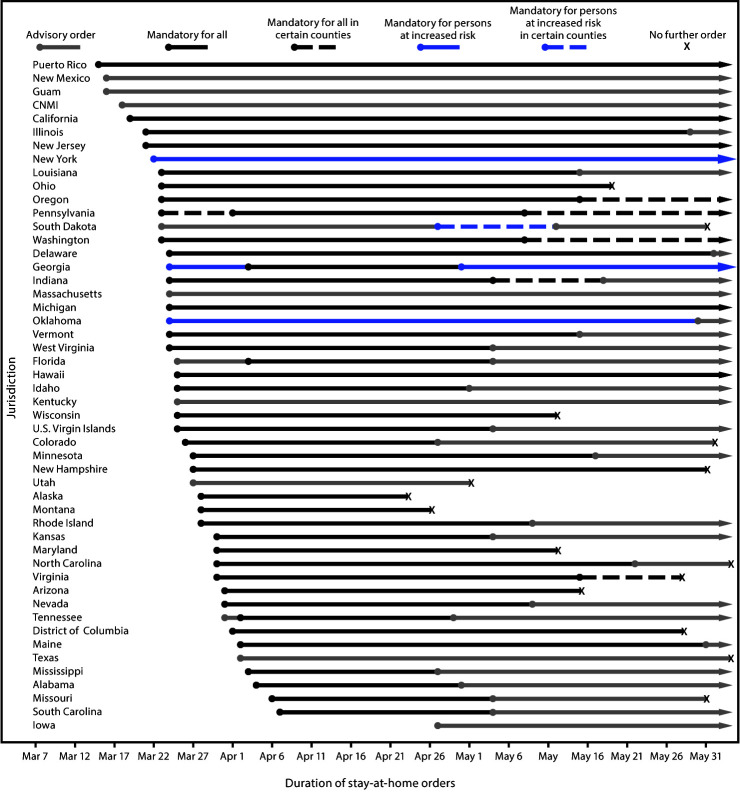
Type and duration of COVID-19 state and territorial stay-at-home orders,* by jurisdiction — United States,† March 1–May 31, 2020 **Abbreviations:** COVID-19 = coronavirus disease 2019; CNMI = Northern Mariana Islands. * Including the type of stay-at-home order implemented, to whom it applied, and the period for which it was in place. ^†^ Jurisdictions that did not issue any orders requiring or recommending persons to stay home during the observation period were not included in this figure. Jurisdictions without any orders were American Samoa, Arkansas, Connecticut, Nebraska, North Dakota, and Wyoming.

Differences in county-level mean population movement during the pre- and postorder periods varied by index date and rural-urban strata (Figure 2). Decreased median population movement was observed in 2,295 (97.6%) of the 2,351 counties for which population movement data were available. Mandatory stay-at-home orders were associated with decreased population movement (i.e., higher median percentage of devices at home) during the 28-day period after the order start date, relative to the baseline 28-day period before the order start date. This relationship was significant in all rural-urban strata (Supplementary Table, https://stacks.cdc.gov/view/cdc/92406). Among the 2,355 counties subject to mandatory stay-at-home orders, 436 (19%) had an order that expired on or before May 3, which is the latest possible expiration date that allows for a 28-day postorder observation period.^¶¶^ Movement significantly increased (i.e., lower median percentage of devices at home) in the period immediately after the expiration or lifting of orders in all rural-urban strata.

**FIGURE 2:**
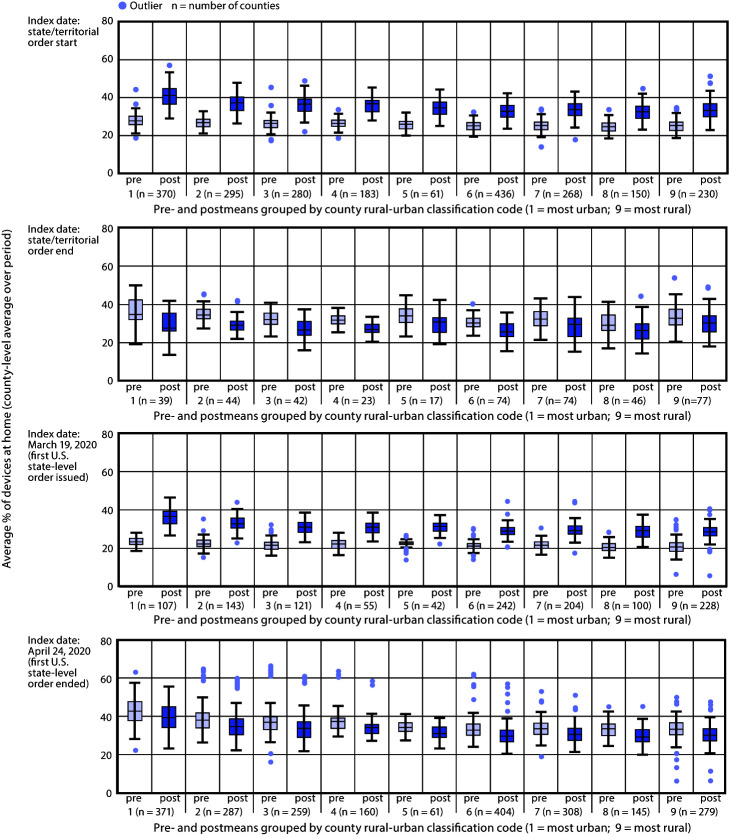
Distribution of county-level mean percentage of mobile devices at home pre- and postindex date periods (relative to the start and end of stay-at-home orders), by rural-urban classification — United States, March 1–May 31, 2020

The 14-day period immediately after the first state stay-at-home order was issued in the United States was associated with a significant decrease in movement in all rural-urban strata relative to the 14-day period immediately preceding its implementation.*** The period after the first state relaxed a stay-at-home order was associated with increased population movement at the strata level among states or territories that had not relaxed a stay-at-home order in the same period.^†††^

## Discussion

Based on location data from mobile devices, in 97.6% of counties with mandatory stay-at-home orders issued by states or territories, these orders were associated with decreased median population movement after the order start date, relative to the period before the order was implemented. Reduced population movement helps prevent close contact among persons outside the household, potentially limiting exposure to persons infected with SARS-CoV-2. This suggests that stay-at-home orders can help protect the public’s health by limiting potential exposure to SARS-CoV-2 and reducing community transmission of COVID-19.

The implementation of stay-at-home orders might affect population movement differently depending on when and where orders are issued and to whom they apply. The observed decrease in population movement after the implementation of the first state-issued mandatory stay-at-home order in California suggests that the implementation of certain public health policies might influence behaviors in other areas, in addition to persons directly subject to the action. However, this observation occurred in the context of other variables, which might have influenced behaviors, including the declaration of COVID-19 as a pandemic, declaration of national or state emergencies, media attention to fatalities and increased demands on hospitals, gathering bans, closures of schools and businesses, and cancellation of sporting events.

Increases in population movement were evident among counties in jurisdictions where stay-at-home orders were lifted, as well as in other communities as orders began to lift nationwide. Such increases might be driven in part by persons resuming preorder movement behaviors in response to the lifting of orders where they lived, or in response to perceived reduced risk associated with the lifting of orders elsewhere. Many other factors might have also played a role, and additional studies are needed to determine which factors caused population movement to increase across jurisdictions after the first state stay-at-home order ended.^§§§^

Further research is needed to assess the impact of reduced population movement and other community mitigation strategies on the spread of COVID-19. For example, understanding the relationship between stay-at-home orders in contiguous counties and movement might explain how same-state and neighboring-state policy changes can affect public health by mitigating or exacerbating external environmental and social factors affecting population movement.^¶¶¶^ As the pandemic continues and jurisdictions consider reimplementing mitigation policies, additional studies are needed to assess the impact of reissuing stay-at-home orders.

The findings in this report are subject to at least five limitations. First, although relative device coverage largely correlates with U.S. population density, some regions or demographic groups might be over- or underrepresented.**** Second, persons might have multiple mobile devices and might not take certain devices with them when they leave the home (e.g., tablets) or might take multiple devices with them simultaneously (e.g., phones and smart watches). Third, although the clustered Wilcoxon signed rank test is used with counties as clusters because each county’s median pre- and postorder values are paired comparisons rather than independent observations, potential spatial dependence among counties is not addressed. Fourth, this report does not assess whether population movement was affected by nationwide protests during the observation period.^††††^ Finally, this report analyzes the relationship between stay-at-home orders and population movement and does not assess the complex relationship between stay-at-home orders and illness incidence rates or deaths.

Mandatory stay-at-home orders can help reduce activities associated with community spread of COVID-19, including population movement and close person-to-person contact outside the household. Mandatory stay-at-home orders were associated with reduced population movement in most counties during the early months of the COVID-19 pandemic, and the relaxation of those orders was associated with increased movement. Although stay-at-home orders might assist in limiting potential exposure to SARS-CoV-2 and have had public support (*7*), such orders substantially disrupt daily life and have resulted in adverse economic impact (*8*). Further studies are needed to assess the timing and conditions under which stay-at-home orders might be best used to protect health, minimize negative impacts, and ensure equitable enforcement of community mitigation policies. These findings can inform public policies to potentially slow the spread of COVID-19 and control other communicable diseases in the future.

**Summary**

**What is already known about this topic?**

Stay-at-home orders are a community mitigation strategy used to reduce the spread of COVID-19 in the United States.

**What is added by this report?**

States and territories that issued mandatory stay-at-home orders experienced decreased population movement in most counties. The period after the first state relaxed a stay-at-home order was associated with increased population movement in states or territories that had not relaxed a stay-at-home order in the same period.

**What are the implications for public health practice?**

Stay-at-home orders can reduce activities associated with community spread of COVID-19, including population movement and close person-to-person contact outside the household. These findings can inform future public policies to reduce community spread of COVID-19.

## Footnotes

* American Samoa, Guam, Northern Mariana Islands, Puerto Rico, and U.S. Virgin Islands.

† Coding of orders was based on the legal language in each state or territorial order; this analysis did not assess order enforcement, public perception, or the impact of other mitigation policies. An order was coded mandatory if it contained language requiring persons to stay home (e.g., persons “shall,” “must,” or “are directed to”) or advisory or recommendation if it contained permissive language suggesting persons stay home (e.g., persons “should,” “are encouraged to,” or “are urged to”). Orders were coded mandatory only for persons in certain areas of the jurisdiction if the order expressly required persons in certain areas (e.g., counties) to stay home but did not require persons in other areas to stay home. Orders were coded mandatory only for persons at increased risk in the jurisdiction if they expressly required persons who meet certain high-risk criteria (e.g., aged >65 years or those with chronic medical conditions) to stay home while permitting others to leave their homes.

§ Given the set of state-issued mandatory stay-at-home orders described, and any particular state order associated with state *s* that goes into effect at time *t*, one can define pre- and postorder windows for each county, *c* in *s.* A given county, *c'*s preorder window will contain observed values for the movement metric of interest, *m*, during the *n*-day period before the order index date, *t*, and the postorder window will contain observed values for *m* during the *n*-day period after *t*. In this way, each county's preorder window serves as a county- and COVID-specific baseline, in that (for sufficiently small values of *n*), the values observed during this period reflect both county-specific invariants and the impact of the pandemic on behavior in the absence of state- or territory-issued community mitigation policies.

¶ The U.S. Department of Agriculture’s Rural-Urban Continuum Codes are used to stratify counties in this analysis. https://www.ers.usda.gov/data-products/rural-urban-continuum-codes/documentation/.

** https://arxiv.org/abs/1706.03409v1.

†† 45 C.F.R. part 46, 21 C.F.R. part 56; 42 U.S.C. Sect. 241(d); 5 U.S.C. Sect. 552a; 44 U.S.C. Sect. 3501 et seq.

§§ https://www.wicourts.gov/sc/opinion/DisplayDocument.pdf?content=pdf&seqNo=260868.

¶¶ The comparison of movement data while orders were in effect versus after expiration excludes counties located in the 14 states and territories that never implemented a mandatory stay-at-home order during the observation period, as well as counties in 35 states and territories with mandatory orders that expired after May 3, or were still in place as of May 31, 2020, because bifurcation of county-level population movement data into 28-day pre- and postindex-date windows is not possible in such cases, given data available at the time of publication. All rural-urban strata were represented in the subset of counties after accounting for the postorder period.

*** This analysis includes 1,242 counties for which population movement data were available and which were located in jurisdictions that never issued a mandatory order or had not issued a mandatory order by the end of the 14-day postorder period and excluded the remaining 1,984 counties in states or territories that enacted an order during this period.

††† This analysis includes 2,274 counties for which population movement data were available and which were located in jurisdictions that never issued a mandatory order or still had a mandatory order in place at the end of the 14-day postorder period and excluded the remaining 952 counties in states or territories that relaxed an order during this period.

§§§ Additional factors that might have played a role include perceived reduced movement-associated risk because of social distancing and use of personal protective equipment, as well as the need to return to work, procure essential goods, seek health care, or exercise, particularly when persons might have suspended such activities at the onset of the pandemic or while under stay-at-home orders.

¶¶¶ Potential confounders include protest activity, COVID-19 incidence rates, and socioeconomic factors.

**** Mobile device data do not include characteristics of persons using these devices; therefore, results are not disaggregated by sociodemographic characteristics, nor do these data account for relative differences in population movement (e.g., number of trips out of the home, social distancing, or method of transportation). Additional information on data and bias correction is available at https://www.safegraph.com/blog/what-about-bias-in-the-safegraph-dataset.

†††† https://www.nytimes.com/article/george-floyd-protests-timeline.html; https://www.nytimes.com/2020/04/18/us/texas-protests-stay-at-home.html.
